# Prolactin: Friend or Foe in Central Nervous System Autoimmune Inflammation?

**DOI:** 10.3390/ijms17122026

**Published:** 2016-12-02

**Authors:** Massimo Costanza, Rosetta Pedotti

**Affiliations:** Department of Clinical Neuroscience, Neurological Institute Foundation IRCCS Carlo Besta, 20133 Milan, Italy

**Keywords:** prolactin, multiple sclerosis, experimental autoimmune encephalomyelitis, neuroinflammation, neuroprotection

## Abstract

The higher prevalence of multiple sclerosis (MS) in females, along with the modulation of disease activity observed during pregnancy and the post-partum period, has suggested a hormonal influence in MS. Even if prolactin (PRL) does not belong to the sex hormones family, its crucial role in female reproduction and lactation has prompted great efforts to understand if PRL could represent a gender factor in the pathogenesis of MS and experimental autoimmune encephalomyelitis (EAE), the animal model for this disease. Extensive literature has documented a remarkable immune-stimulating potential for this hormone, indicating PRL as a disease-promoting factor in MS and EAE. However, recent work has pointed out that PRL is endowed with important neuroprotective and remyelinating properties and has encouraged a reinterpretation of the involvement of this hormone in MS. In this review we summarize both the protective functions that PRL exerts in central nervous system tissue as well as the inflammatory activity of this hormone in the context of autoimmune responses against myelin. Last, we draw future lines of research that might help to better clarify the impact of PRL on MS pathology.

## 1. Introduction

Multiple sclerosis (MS) is a chronic, inflammatory disorder of the central nervous system (CNS) that affects more than 2.5 million people worldwide, representing the most common cause of neurologic disability in the white young adult population [1]. Neurological symptoms include sensory disturbances, limb weakness and paresis, fatigue, and sexual and bladder dysfunctions [1]. MS is commonly believed to originate from a detrimental interaction between genetic and environmental factors, which leads to the establishment of a T helper (Th)1/Th17 cell-driven autoimmune response against myelin in the CNS [2]. Inflammation triggers demyelination and axonal injury, resulting in defective propagation of action potentials through the internodes of nerves (loss of saltatory conduction) and neurological symptoms [3]. The main histological feature of acute MS is represented by the formation of “plaques” in multiple sites within CNS white matter [4]. These areas are infiltrated by peripheral immune cells, such as macrophages, T cells, B lymphocytes and plasma cells, and display evidence of myelin loss and axonal injury [4].

Depending on geographical areas, MS has a sex ratio (female:male) of 2:1 to 3:1 [5]. Women affected by MS experience a substantial reduction of disease activity during the third trimester of pregnancy, and an increased rate of disease attacks in the trimester after delivery [6]. These observations have suggested a hormonal influence on MS pathogenesis. Even if prolactin (PRL) does not belong to the sex hormones family, its crucial role in female reproduction and lactation [7], along with higher serum concentrations found in females vs. males [8], have suggested PRL as a gender factor in MS [9]. The interest toward this hormone in the MS field has also been renewed by a recent debate related to the convenience of breastfeeding (a hyperprolactinemic condition) for MS women. Indeed, two clinical studies have reported that exclusive breastfeeding, a condition when infants are fed solely by breast milk, can actually reduce the risk of relapses during the post-partum period in MS [10,11]. However, other studies were not able to confirm the same results [12,13,14].

Prolactin is a 23 kDa polypeptide hormone mainly secreted by the lactotrophic cells of the anterior pituitary gland, even though other cell types can also release this hormone, including immune cells, adipocytes, mammary and epithelial cells [15]. Transcription of the *PRL* gene is controlled by the pituitary and extrapituitary (also known as superdistal) promoters in humans [15]. The PRL receptor (PRLR) is the only known receptor for PRL and it belongs to the cytokine receptor superfamily, which include receptors for leptin, interleukin (IL)-2, IL-6, and others [16,17]. PRLR binding activates downstream signaling cascades involving JAK/STAT, MAPK and PI3K/Akt intracellular pathways [18]. Broad evidence since the 1970s has demonstrated that PRL can stimulate cells of both innate and adaptive immune systems [19] and therefore PRL has long been considered a potentially detrimental agent in MS and experimental autoimmune encephalomyelitis (EAE), the animal model for this disease. However, more recent papers have highlighted that PRL is unexpectedly endowed with neuroprotective and promyelinating properties, prompting a reconsideration of the role of PRL in MS and EAE.

In this review, we attempt to provide an integrated overview of PRL involvement in MS and EAE, summarizing both the protective functions that PRL exerts on CNS tissue as well as the inflammatory potential of this hormone in the context of autoimmune responses against myelin. Last, we trace future lines of research aimed at further understanding the involvement of PRL in the pathogenesis of MS and EAE.

## 2. Prolactin: A Regenerative Hormone for Central Nervous System Tissue

In recent years a significant amount of work has documented numerous actions mediated by PRL in the CNS. Circulating PRL can cross the blood-brain barrier; however, its transport into the brain does not depend on PRLR but is mediated by a still-unidentified transport molecule [20].

### 2.1. Prolactin Sustains Adult Neurogenesis

Several studies in the last years have demonstrated that PRL can positively affect neurogenesis in several physiological and pathological conditions. PRL is important for pregnancy-stimulated neurogenesis of the female adult brain, a process that probably supports maternal adaptation to offspring [21]. PRLR has been detected on the choroid plexus and on the dorsolateral corner of the subventricular zone (SVZ), one of the neurogenic areas of the adult forebrain [21]. In pregnant *Prlr*^+/−^ mice (*Prlr*^−/−^ mice are sterile), a 50% reduction of PRLR is sufficient to induce a significant reduction of proliferating neural progenitors in the SVZ and a consequent reduction of olfactory bulb interneurons. Interestingly, when adult neurospheres are cultured in vitro in the presence of PRL, the number of differentiated neurons is doubled [21]. PRL was later shown to be required also for paternal recognition of adult offspring, promoting neurogenesis in the SVZ and in the hippocampal dentate gyrus [22]. Another group has demonstrated that direct injection of PRL in the mouse dentate gyrus almost duplicates the number of neurospheres obtained ex vivo [23]. Moreover, PRL-deficient mice display learning and memory deficits which are rescued by administration of PRL into the hippocampus [23]. The impact of PRL on neurogenesis has also been evaluated during chronic exposure to stress, a condition that results in the diminishment of hippocampal neurogenesis. It was found that repeated peripheral administration of PRL to adult mice subjected to chronic stress—4 h of daily immobilization for 21 days—can rescue adult hippocampal neurogenesis [24]. Again, PRL treatment during stress conditions induces a higher percentage of newborn neurons if compared to vehicle-treated mice [24].

### 2.2. Prolactin as a Neuroprotective Factor

The actions of PRL in the CNS are not solely restricted to adult neurogenesis. PRL has been indicated as a promising therapeutic agent for spinal muscular atrophy (SMA), a neurodegenerative disorder characterized by the loss of motoneurons and progressive muscular atrophy [25]. Systemic administration of PRL in a mouse model of severe SMA promotes a drastic improvement of motor functions, associated with a slowdown of weight loss and enhanced survival [25]. In a model of kainic acid-induced epilepsy, rats treated with PRL are protected from cell loss in the dorsal hippocampus and exhibit diminished seizure behavior if compared to vehicle-treated animals [26]. The neuroprotective effects of PRL have been confirmed in another model of neurotoxicity obtained in vitro by exposing primary rat hippocampal neurons to glutamate. PRL counteracts glutamate-induced cell death, and this effect is abolished when neurons are treated with antisense oligonucleotides for PRLR [27]. Another work has observed that PRLR-deficient mice are affected by photoresponsive dysfunction, suggesting that PRL is important for retinal activity and visual perception [28]. In a model of retinal degeneration obtained by prolonged exposure of rats to bright light, hyperprolactinemia mostly prevents retinal degeneration and dysfunction. In this model, PRL has been suggested to reinforce the neurotrophic support provided by retinal glial cells and to lessen gliosis [28].

### 2.3. Promyelinating Effects of Prolactin

Oligodendrocytes are also positively affected by PRL stimulation. Gregg and coworkers have shown that murine pregnancy promotes the increase of oligodendrocyte precursor cells (OPCs) and myelinated axons in the female adult brain [29]. Pregnant mice also display enhanced remyelinating capacity following acute demyelinating injury triggered by the injection of the detergent lysolecithin in the spinal cord. This enhanced white matter plasticity is significantly impaired in pregnant *Prlr*^+/−^ mice. Moreover, PRL administration supports myelin repair in virgin females receiving lysolecithin lesions [29]. Given its remyelinating capacity, PRL has also been proposed as a therapeutic agent for MS.

## 3. Prolactin and Central Nervous System Autoimmune Responses

### 3.1. The Immune-Stimulating Properties of Prolactin

Along with homeostatic and regenerative actions in the CNS, PRL is also endowed with immune-stimulating functions, even though most evidence relies on in vitro experiments. Several immune cells involved in the autoimmune attack against the CNS occurring in MS and EAE can be stimulated by PRL. Macrophages exposed to PRL secrete higher amounts of reactive oxygen species, and inflammatory cytokines (e.g., interferon (IFN)-γ and IL-12) and chemokines (e.g., macrophage inflammatory protein (MIP)-1α and monocytes chemoattractant protein (MCP)-1) [30,31,32]. PRL also supports the maturation of dendritic cells derived from monocytic precursors, promoting the expression of MHC class II and CD80/86 co-stimulatory molecules [33,34]. PRL has been proposed as an autocrine factor sustaining survival and proliferation of human T cells [35,36]. On the contrary, no *Prl* transcript or protein have been detected in purified CD4^+^ T cell cultures of mouse origin [37]. In this regard, it is important to consider that a single promoter drives *Prl* expression in rodents, while an additional “extra-pituitary” promoter has been described in humans [38]. This difference in the organization of *PRL* transcription should be taken into account when translating concepts from experimental models to humans, as a more complex regulation of PRL production might occur in humans. Regulatory T cells (Treg) represent a T cell subset with immune-suppressive functions and their expansion is severely impaired in MS patients [39]. Interestingly, human Treg cells constitutively express PRLR and their suppressive capacity is significantly abated when they are treated in vitro with PRL [40]. The activity of PRL on B and plasma cells has been extensively investigated in systemic lupus erithematosus (SLE), an autoimmune disorder characterized by deregulated humoral immune responses [41]. In different experimental models, it has been observed that hyperprolactinemia contributes to the break of B cell tolerance, leading to enhanced maturation of B cells, increased levels of circulating autoantibodies and immunoglobulin deposition [42,43,44,45]. Human B cells increase *PRLR* expression and PRL secretion following stimulation [46].

### 3.2. Prolactin and Multiple Sclerosis

Several groups have investigated a possible correlation between PRL levels in body fluids and MS. A recent study has reported that female MS patients have significantly higher PRL levels in serum and cerebrospinal fluid if compared to healthy donors, male MS subjects or patients with clinically-isolated syndrome [47]. However, PRL concentrations were not correlated with disease activity or duration [47]. Conversely, Moshirzadeh and colleagues detected increased serum PRL levels in both female and male relapsing-remitting MS patients during relapses, if compared to healthy donors [48]. Another work has found a positive correlation between plasma PRL levels and white matter volume in MS subjects [49]. Overall, there is no consensus on the association between MS and hyperprolactinemia [50]. An interesting clinical report has documented the case of a 32-year-old MS patient who developed the first white matter lesions in association with the development of a PRL-secreting adenoma, which approximately doubled the serum PRL levels [51]. The adenoma was excised, but 12 years later the patient suffered two additional disease attacks, in concomitance with adenoma recurrence [51]. In a recent work, the effect of PRL on B cells isolated from MS patients has been explored [46]. PRL significantly enhances the number of cells secreting antibodies directed against myelin oligodendrocyte glycoprotein (MOG) in MS subjects and upregulates the expression of B cell activating factor (BAFF) and the anti-apoptotic molecule Bcl2, via a Jak2/STAT-dependent pathway [46]. Moreover the threshold of B cell activation is importantly reduced after PRL exposure. Authors also reported a negative correlation between PRL serum levels and the number of apoptotic B cells isolated from MS patients [46]. A couple of studies have explored the therapeutic efficacy of lowering PRL levels in MS by using bromocriptine (BCR), a D2 dopaminergic agonist that blocks PRL secretion from the pituitary gland. In the first open-label study, authors reported no efficacy of BCR in dampening disease activity [52]; a second case report described the resolution of paroxysmal symptoms in a single patient after BCR administration [53]. However, the effects of BCR are not solely restricted to those on PRL [54,55], and therefore there is no conclusive indication on the potential efficacy (or toxicity) of targeting PRL in MS.

### 3.3. Prolactin and Experimental Autoimmune Encephalomyelitis

Attempts to lessen MS activity by administration of BCR were based on results in EAE, a widely used animal model of Th1/17-mediated autoimmune demyelination of the CNS. Active EAE is mostly induced in susceptible strains of rats or mice, by immunization with immunodominant epitopes of myelin antigens supplemented with adjuvants [56]. EAE generally develops as a flaccid paralysis starting from the tail and progressing to hind and forelimbs [57]. BCR was found to alleviate the clinical manifestation of EAE in rats and to dampen encephalitogenic T cell responses [58,59]. However, as previously discussed, BCR therapeutic effects might also be independent of PRL reduction. Others and we have recently characterized the development of chronic EAE in *Prlr*^−/−^ and *Prl*^−/−^ mice, elicited by immunization with MOG peptide 35–55 (MOG_35–55_). We reported that mice lacking PRLR or PRL develop chronic EAE with a modestly but significantly delayed onset and a disease severity comparable to control mice [37]. The retardation in the presentation of clinical symptoms observed in *Prlr*^−/−^ and *Prl*^−/−^ mice is associated with a mild delay in the development of Th1 and Th17 autoimmune responses against myelin in draining lymph nodes [37]. This work has suggested that the absence of PRL or its receptor during chronic EAE leads to a minor deficit in the CNS autoimmune demyelinating response, which can be easily counterbalanced by other factors. Therefore, PRL does not play a crucial role in this model. Consistent with our results, another work has described that spleen cells from EAE mice stimulated with myelin antigen and exposed to PRL display a dose-dependent increase of the proliferative response [60]. In this work, authors also explored the therapeutic potential of a combination therapy composed of PRL and IFN-β for chronic EAE, in order to exploit the remyelinating properties of PRL and control autoimmune responses with IFN-β at the same time. They found that treatment of EAE mice with PRL and IFN-β induces an amelioration of disease symptoms and a reduction of immune cell infiltration in the spinal cord, if compared to vehicle-treated mice [60]. However, this treatment showed efficacy only when mice developed mild signs of disease, while it was not effective in case of severe EAE [60].

## 4. Conclusions and Future Perspectives

**Figure 1:**
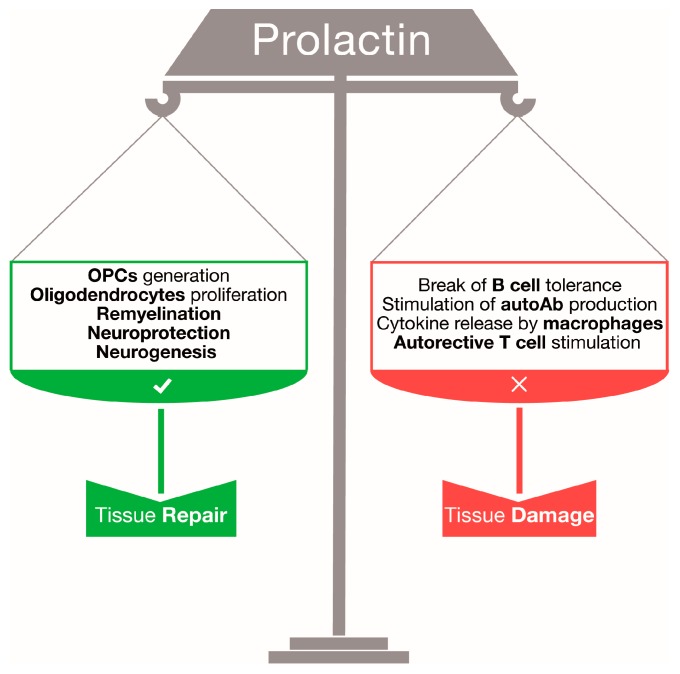
The potential duality of prolactin (PRL) in central nervous system (CNS) autoimmunity. PRL might promote CNS repair by influencing oligodendrocyte progenitor cells (OPC) generation and oligodendrocyte proliferation [29], and favoring remyelination [29], neuroprotection [25,26,27,28] and neurogenesis [21,22,23,24]. However, PRL might also promote CNS damage by breaking B cell tolerance [42,43,44,45,46], sustaining autoantibody (autoAb) production [45] and macrophage release of cytokines [30,31,32] and stimulating autoreactive T cells [37,40].

Data discussed in this review suggest that PRL might play dual and opposing functions in both MS and EAE. Its effects are likely to be the result of a fine balance between protective actions on CNS tissue and stimulation of the immune system. On the one hand, PRL might provide regenerative signals for neurons, oligodendrocytes and adult neural stem/progenitor cells, and thus support CNS repair; on the other hand, PRL stimulation of peripheral immune cells, in particular T and B lymphocytes, might sustain autoimmune responses and negatively impact on disease pathology (Figure 1).

The development of MOG_35–55_-induced EAE in non-pregnant mice is not substantially influenced by the absence of PRL or its receptor [37]. Given that B cells are dispensable for disease induction in this model [61] and given the important effects exerted by PRL on B cell maturation and functions, it might be interesting to explore if PRL/PRLR deficiency impacts on disease development in B cell-dependent EAE models, such as recombinant MOG-induced EAE [61].

From a therapeutic perspective, the dual nature of this hormone suggests great caution when trying to manipulate PRL levels in MS. It is also possible to speculate that PRL depletion or administration might be beneficial, depending on the MS form and/or stage. Also, no indication on breastfeeding can be drawn by data produced so far in experimental models. In this regard, the interpretation of results obtained in mouse models in the context of human immune responses and autoimmunity is complicated by the relatively recent discovery in humans of a cleaved 16 kDa isoform of PRL. This PRL isoform has different properties from the full 23 kDa PRL, exerting anti-angiogenic functions and mediating cardiomyopathy in the post-partum period [62]. It must also be kept in consideration that several physiological functions are differentially regulated by PRL in rodents and humans; as additionally, a different pattern of PRL expression exists between these species [15]. For example, while PRL is luteotropic and has a fundamental role in the establishment of pregnancy in rodents, it seems not to exert crucial functions in human reproduction, with the exception of lactation [15]. Also, the profile of PRL secretion during gestation is significantly different between rodents and humans [15]. A major diversity between these species is the presence of several extra-pituitary sites of PRL production in humans, such as decidual and breast tissues, adypocytes, immune cells and the prostate [38,63]. In many of these sites, a “superdistal” promoter controls the expression of an alternative *PRL* transcript, which was first identified in human lymphocytes and endometrial tissue [64,65]. The existence of extra-pituitary locations of PRL production in humans implies a complex, tissue-specific regulation of PRL functions, which can mediate autocrine and paracrine effects in different compartments. Of note, in a transgenic mouse model carrying the human *PRL* gene and all its regulatory elements, the human, but not mouse, *PRL* transcript was detected in lymphoid organs, such as the spleen and thymus [66]. In order to help fill the biological gap between rodent and human PRL, it might be useful to investigate the role of this hormone on the development of EAE induced in non-human primates, such as marmosets (*Callithrix jacchus*) [67], or in a recently generated humanized mouse model, in which the mouse *Prl* gene was replaced with human *PRL* [66].

In conclusion, further studies are still necessary to better clarify how PRL influences autoimmune responses of the CNS and whether PRL might represent a target of therapy in these disorders.

## Abbreviations

BCR	Bromocriptine
CNS	Central Nervous System
EAE	Experimental Autoimmune Encephalomyelitis
IFN	Interferon
IL	Interleukin
MOG	Myelin Oligodendrocyte Glycoprotein
MS	Multiple Sclerosis
OPC	Oligodendrocyte Progenitor Cell
PRL	Prolactin
Treg	Regulatory T cells
